# A survey of ocular pathology in Warmblood horses in South Africa

**DOI:** 10.1111/evj.14427

**Published:** 2024-11-13

**Authors:** Ramona Allen, Antony D. Goodhead

**Affiliations:** ^1^ Faculty of Veterinary Science University of Pretoria Onderstepoor Pretoria South Africa

**Keywords:** cataract, chorioretinopathy, eye disease, horse, warmblood

## Abstract

**Background:**

Warmblood horses are a popular breed around the world for equestrian sports. Previous studies have investigated ocular findings in other breeds of horses; however, no studies exist for the Warmblood breed.

**Objectives:**

To determine the prevalence of ocular abnormalities in a convenience sample of Warmblood horses in South Africa and to determine if the prevalence of lens and chorioretinal lesions increase with age.

**Study design:**

Descriptive, observational study.

**Methods:**

Warmblood horses underwent a full ophthalmic examination which included a Schirmer tear test (STT), tonometry, fluorescein dye testing, slit lamp biomicroscopy and indirect ophthalmoscopy. Age was categorised into three groups namely <8 years old, 8–13 years old and 14+ years old for statistical analysis. Prevalence of lens and chorioretinal lesions were compared between age categories.

**Results:**

One hundred and four horses (208 eyes) were examined. The age range was 5 months to 30 years (mean 11 years, standard deviation [SD] 6 years). Ocular pathology was noted in 125 eyes (60.1%) and 79 horses (76%). The highest number of lesions were noted in the choroid and retina, iris and lens. Chorioretinal lesions were seen in 100 eyes (48.1%) and in 65 horses (62.5%). Iridial lesions were seen in 19 eyes (9.1%) and 12 horses (11.5%). Cataracts were seen in 19 eyes (9.1%) and in 13 horses (12.5%). The presence of total chorioretinal lesions (eye level [*p* = 0.002]; horse level [*p* = 0.004]), focal chorioretinal lesions (eye level [*p* = 0.004]; horse level [*p* = 0.008]) and cataract (eye [*p* = 0.03]; horse level [*p* = 0.02]) were all shown to statistically increase with age.

**Main limitations:**

A small sample size and limited geographic area.

**Conclusions:**

There was a high prevalence of ocular pathology in this population of Warmblood horses in South Africa. This reiterates the importance of an ocular examination as a part of routine health checks, as well as during prepurchase examinations.

## Résumé

**Contexte:**

Les chevaux Warmblood constituent une race populaire pour les sports équestres au niveau mondial. Alors que d'autres études ont investigué les trouvailles oculaires chez les chevaux d'autres races, il n'existe aucune étude de ce type pour la race Warmblood.

**Objectifs:**

Déterminer la prévalence des anomalies oculaires dans un échantillon de convenance comprenant des chevaux Warmblood en Afrique du Sud et si la prévalence des lésions du cristallin et choriorétinales augmentent avec l'âge.

**Type d'étude:**

Descriptive, observationnelle.

**Méthodes:**

Des chevaux Warmblood ont été soumis à un examen oculaire complet incluant un test de Schirmer, tonométrie, test à la fluorescéine, microscope à lampe à fente, et ophtalmoscopie indirecte. Les chevaux ont été catégorisé par âge en trois groupes soit <8 ans, 8–13 ans et plus de 14 ans pour les analyses statistiques. La prévalence des lésions du cristallin et choriorétinales ont été comparées par catégorie d'âge.

**Résultats:**

Cent quatre chevaux (208 yeux) ont été examinés. L'étendue d'âge était de 5 mois‐30 ans (moyenne 11 ans, déviation standard 6 ans). Des pathologies oculaires ont été notées dans 125 yeux (60.1%) et 79 chevaux (76%). Le nombre de lésions le plus élevé était dans la choroïde et la rétine, l'iris et le cristallin. Des lésions choriorétinales étaient présentes dans 100 yeux (48.1%) et chez 65 chevaux (62.5). Des lésions de l'iris étaient présentes dans 19 yeux (9.1%) et chez 12 chevaux (11.5%). Des cataractes étaient présentes dans 19 yeux (9.1%) et chez 13 chevaux (12.5%). La présence totale de lésions choriorétinales (niveau de l'œil *p* = 0.0023; niveau du cheval *p* = 0.0042), de lésions choriorétinales focales (niveau de l'œil *p* = 0.004; niveau du cheval *p* = 0.008) et de cataractes (niveau de l'œil *p* = 0.03; niveau du cheval *p* = 0.02) étaient toutes significativement augmentées avec l'âge.

**Limites Principales:**

Petite taille d'échantillon et région géographique limitée.

**Conclusion:**

La prévalence de pathologies oculaires est. élevée dans cette population de chevaux Warmblood en Afrique du Sud. Ceci renforce l'importance de l'examen oculaire comme partie intégrante des examens de santé de routine, de même que lors d'examens d'achat.

## 1 INTRODUCTION

Warmblood horses are a popular breed worldwide for equestrian sports such as showjumping and dressage, and generally compete over a longer period of time in comparison to equids used for other equestrian sports such as racing. Ocular abnormalities could therefore significantly reduce performance and even be vision‐threatening. Furthermore, when selecting breeding horses, little emphasis is placed on ophthalmic examination compared with other traits such as conformation, gaits and performance.^1^

There are no studies on general ophthalmic examination of the Warmblood horse, in contrast to other breeds such as the Thoroughbred, Lipizzaner, Rocky Mountain Horses, Polish Arabian horses, Exmoor Pony, Old Kladruber Horses, Icelandic horse and miniature horses.^2^, ^3^, ^4^, ^5^, ^6^, ^7^, ^8^, ^9^, ^10^, ^11^ Our aim was to therefore investigate the prevalence of and document types of ocular pathology in a convenience sample of Warmblood horses in South Africa in horses without known ocular disease. Outcomes from the study may then influence advice on ophthalmic examination of horses, especially those used in a competitive setting. This would include horses which are intended for breeding purposes, as certain ocular conditions may possibly have a heritability factor.^12^

## 2 MATERIALS AND METHODS

### 2.1 Study population

Warmblood horses housed in various liveries, riding schools and stud farms in the Gauteng and North West Provinces of South Africa were examined. Convenience sampling was used due to the location and accessibility of horses at various locations. A detailed consent form and study rationale were provided to the registered owners or managers prior to any examination. All Warmblood horses, amenable to an ocular examination at each location were included in the current study. Individuals with a history of ocular disease were excluded, which resulted in a single horse being excluded due to a prior ocular trauma which resulted in enucleation. Horses that required sedation for examination were also excluded.

### 2.2 Ophthalmic examination

All horses were examined without sedation or regional anaesthesia in a darkened stable by both a board‐certified veterinary ophthalmologist and a University of Pretoria veterinary ophthalmology resident. Ophthalmic findings together with age, sex, country of birth and activity type were all recorded at time of examination. A general ‘hands off’ examination was first performed to assess globe size, position and symmetry. Prior to assessing the ocular structures, a STT‐1 (STT; Merck Animal Health) and rebound tonometry using the horse setting (TonoVet®, iCare) were performed. Following this direct and consensual pupillary light reflex, menace response and dazzle reflex were tested. Care was taken to direct the menace response at both the nasal and temporal fields. Slit lamp biomicroscopy was performed on the ocular adnexa and anterior segment using a slit lamp biomicroscope (PSL, Keeler). Mydriasis was achieved using 1% tropicamide hydrochloride (Mydriacyl, Alcon) for indirect ophthalmoscopy of the posterior segment of the globe which was performed using an indirect headset (Vantage Plus, Keeler) and panretinal 2.2D condensing lens (Panretinal 2.2, Volk). Fluorescein stain (Minims, Bausch and Lomb) was applied last to assess for any corneal ulceration.

### 2.3 Data analysis

Data collected were recorded on data collection sheets and captured using Apple Numbers version 6.2 (Apple Inc.). Statistical analysis was done using Stata 15.1 (StataCorp) and SPSS 29.0 (IBM Corp.). Prevalence of each lesion was calculated at eye level and at the horse level. Age was categorised into three groups based on tertiles, namely <8 years old (38 horses); 8–13 years old (36 horses); 14+ years old (30 horses) for statistical analysis and was used to assess if lens and chorioretinal lesions increased with age. Prevalence of cataracts and chorioretinal lesions were compared between age categories using logistic regression with robust standard errors to accommodate for clustering of eyes within horses. Statistical comparison of lesions between age categories are versus a baseline group. Both IOP and STT were compared between left and right eyes and between age categories using linear mixed models to account for clustering of eyes within horses. Significance was assessed at *p* < 0.05.

## 3 RESULTS

A total of 104 Warmblood horses (208 eyes) were examined with a mean age of 11 years (standard deviation [SD] 6 years) and a median age of 9 years (interquartile [IQR] range 9–15 years). The sex distribution was eight stallions (7.7%), 33 mares (31.7%) and 63 geldings (60.6%). Determining breed lineage was beyond the scope of this study; however, country of birth was recorded with most horses born in South Africa; however, other countries represented included Namibia, the Netherlands, Germany, Belgium and Sweden. The vast majority of horses were used for either showjumping or dressage.

**FIGURE 1:**
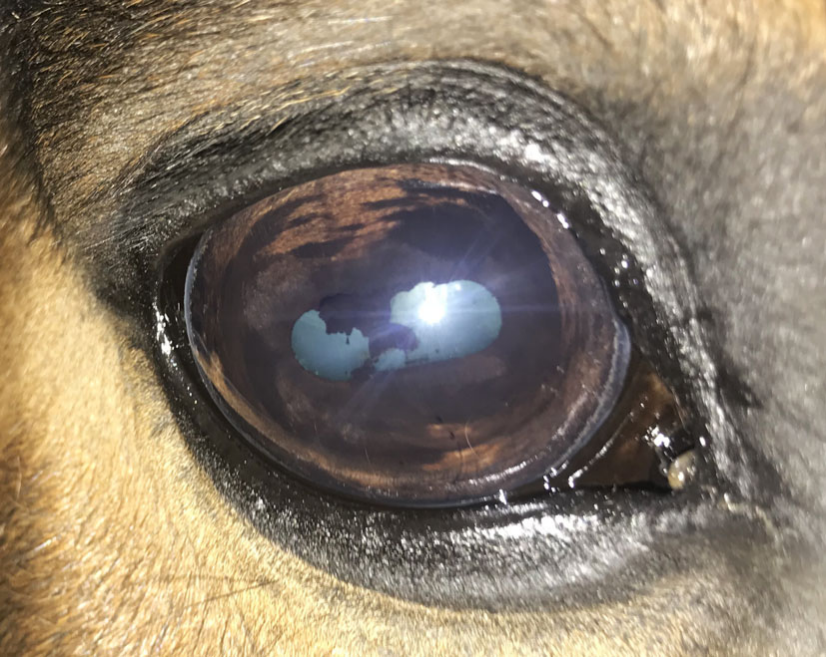
Eye with multiple defects including iris hyperpigmentation, torn, atrophic granula iridica, posterior synechiae and cataract.

All horses in this study were visual bilaterally as assessed by a positive menace response for both the nasal and temporal visual fields. The dazzle reflex was also positive in all eyes. The direct and consensual pupillary light reflex was positive in all eyes, with the exception of one eye, which had multiple anterior segment defects including posterior synechiae (Figure 1).

**FIGURE 2:**
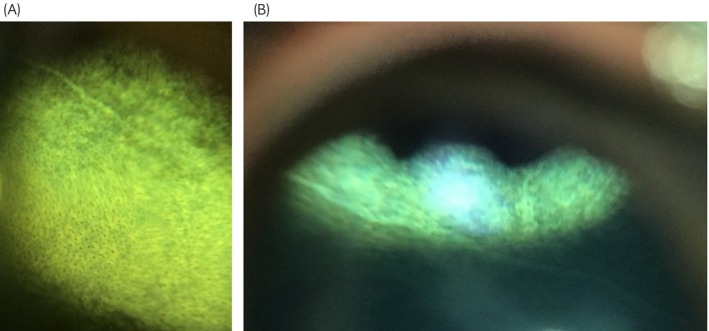
(A and B) Linear, hyperreflective streaks in the tapetal fundus.

Findings were categorised based on the part of the eye affected and the greatest number of lesions were found in the choroid and retina, followed by the iris and then the lens. Table 1 provides a detailed analysis of the ocular lesions identified. Ocular pathology was noted in 125 eyes (60.1%) and 79 horses (76%). A total of 100 eyes (48.1%) and 65 horses (62.5%) had chorioretinal lesions. Focal ‘bullet hole’ chorioretinal lesions were seen in 88 eyes (42.3%) and in 55 horses (52.9%). Non‐peripapillary chorioretinal lesions were seen in 14 eyes (6.7%) and in 12 horses (11.5%). Peripapillary ‘butterfly’ chorioretinal lesions were seen in seven eyes (3.4%) and in six horses (5.8%). In three eyes (1.4%) of three horses (2.9%) linear, horizontal, hyperreflective streaks across the tapetum were noted and termed ‘linear chorioretinopathy’ (Figure 2A,B). The presence of any chorioretinal lesions were more likely with increasing age at both the eye (*p* = 0.002) and horse level (*p* = 0.004, Table 2). Neither peripapillary (eye level *p* = 0.7, horse level *p* = 0.6) nor non‐peripapillary (eye level *p* = 0.06, horse level *p* = 0.052) chorioretinal lesions were associated with age while focal chorioretinal lesions were more likely with increasing age at both the eye (*p* = 0.004) and horse level (*p* = 0.008).

**TABLE 1:** Summary of ocular findings in 208 eyes of Warmblood Horses showing the number of affected eyes (*N*) with percent (%) and 95% confidence interval (CI).

Structure	Abnormality	*N*	%	95% CI
Eyelid	Fibrosis	2	1.0	0.1–3
	Chalazion	1	0.5	0.01–2.7
	Eyelid mass	2	1.0	0.1–3.4
Conjunctiva	Conjunctivitis	12	5.8	3.0–9.9
	Conjunctival follicles	2	1.0	0.1–3.4
	Conjunctival mass	1	0.5	0.01–2.7
Cornea	Corneal fibrosis	2	1.0	0.1–3.4
	Linear keratopathy	1	0.5	0.01–2.7
Iris	Granula iridica hyperplasia	1	0.5	0.01–2.7
	Torn granula iridica	1	0.5	0.01–2.7
	Iris hyperpigmentation	16	7.7	4.5–12.2
	Posterior synechiae	1	0.5	0.01–2.7
	Iris cysts	3	1.4	0.3–4.2
Lens	Total cataracts	19	9.1	5.6–13.9
Vitreous	Vitreal degeneration	8	3.9	1.7–7.4
	Vitreal herniation	1	0.5	0.01–2.7
	Asteroid hyalosis	4	1.9	0.5–4.9
Choroid and retina	Total chorioretinopathy	100	48.1	41.1–55.1
	Focal chorioretinopathy	88	42.3	35.5–49.3
	Peripapillary chorioretinopathy	7	3.4	1.4–6.8
	Non‐peripapillary chorioretinopathy	14	6.7	3.7–11.0
	Linear chorioretinopathy	3	1.4	0.3–4.2
Optic nerve	Optic nerve head mass	1	0.5	0.01–2.7

**TABLE 2:** Results of logistic regression examining the effect of age on the presence of any chorioretinal (CR) lesions and non‐papillary (non‐PP), peripapillary and focal chorioretinopathies at the horse and eye levels with odds ratios (OR), 95% confidence intervals (CI) and *p* values.

	Horse level for 104 horses					Eye level for 208 eyes				
	Any CR					Any CR				
Age	Yes	No	OR	95% CI	*p*	Yes	No	OR	95% CI	*p*
<8 years	16	22	Ref.		0.004	23	53	Ref.		0.002
8–13 years	26	12	3.0	1.2–7.6	0.2	40	36	2.6	1.2–5.6	0.02
>13 years	23	5	6.3	2.0–20.2	0.01	37	19	4.5	1.9–10.6	<0.001
Totals	65	39				100	108			

	Horse level for 104 horses					Eye level for 208 eyes				
	Non‐PP					Non‐PP				
Age	Yes	No	OR	95% CI	*p*	Yes	No	OR	95% CI	*p*
<8 years	3	34	Ref.		0.052	4	72	Ref.		0.06
8–13 years	2	36	0.6	0.1–4.0	0.6	2	74	0.5	0.08–3.1	0.5
>13 years	7	21	3.8	0.9–16.2	0.07	8	48	3	0.7–12.9	0.1
Totals	12	92				14	194			

	Horse level for 104 horses					Eye level for 208 eyes				
	Peripapillary					Peripapillary				
Age	Yes	No	OR	95% CI	*p*	Yes	No	OR	95% CI	*p*
<8 years	2	36	Ref.		0.6	2	74	Ref.		0.7
8–13 years	2	36	1.0	0.1–7.3	1.0	2	74	1.0	0.1–7.1	1.0
>13 years	2	26	2.1	0.3–13.0	0.5	3	53	2.1	0.3–16.1	0.5
Totals	6	98				7	201			

	Horse level for 104 horses					Eye level for 208 eyes				
	Focal					Focal				
Age	Yes	No	OR	95% CI	*p*	Yes	No	OR	95% CI	*p*
<8 years	13	25	Ref.		0.01	19	57	Ref.		0.004
8–13 years	22	16	2.6	1.04–6.7	0.04	35	41	2.6	1.1–5.9	0.03
>13 years	20	8	4.8	1.7–13.9	0.004	34	22	4.6	1.8–11.7	0.001
Totals	55	49				88	120			

*Note*: There were no significant differences in any of the chorioretinal lesions between the oldest horses compared with 8–13 year old horses at the horse or eye level (*p* > 0.2).

**FIGURE 3:**
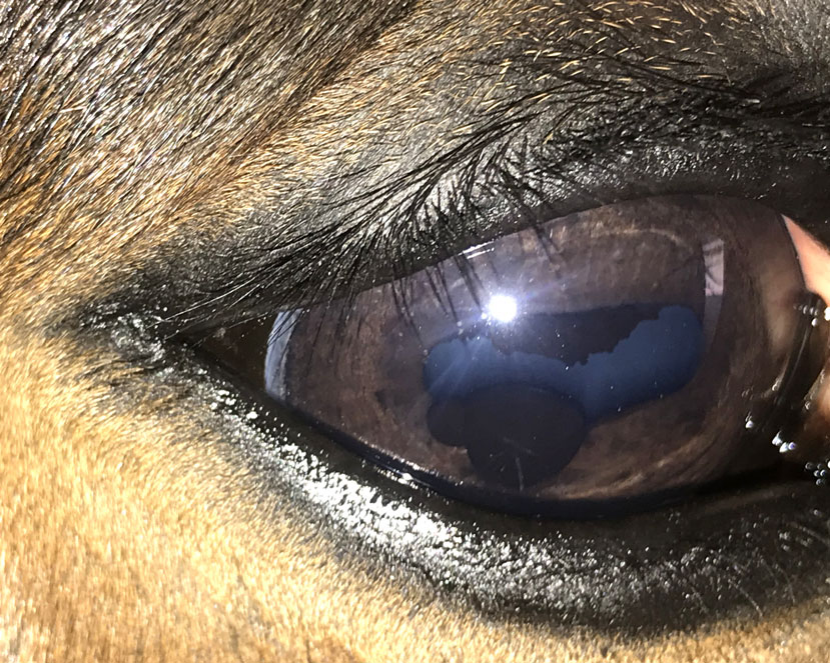
Two iris cysts on the lower pupillary margin.

Lesions of the iris were the second most common type of lesion documented overall with 19 eyes (9.1%) and 12 horses (11.5%) affected. Iris pathology included iris hyperpigmentation which was seen in 16 eyes (7.7%), iris cysts in three eyes (Figure 3) as well as hyperplasia of the granula iridica in one eye. A single eye had a torn granula iridica, hyperpigmentation and posterior synechiae from a presumed previous inflammatory episode.

Cataract formation was noted in 19 eyes (9.1%) and 13 horses (12.5%) all of which were considered incipient cataracts (Table 3). One horse did have a more extensive anterior capsular cataract associated with torn granula iridica, hyperpigmentation and a posterior synechiae due to a presumed traumatic episode; however, fundoscopy was still possible following pharmacologic dilation. The most common cataract noted was anterior capsular followed by axial (floriform) cataract.^13^ Cataracts were more likely to occur with increasing age at both the eye (*p* = 0.03) and horse level (*p* = 0.02, Table 4).

**TABLE 3:** Cataract types identified in 19 eyes of 13 horses by number (*N*) of eyes affected with percent (%) with 95% confidence intervals (CI).

Cataract type	*N*	%	95% CI
Axial (floriform)	6	2.9	1.1–6.2
Anterior capsular^a^, ^b^	8	3.9	1.7–7.4
Anterior polar^b^	3	1.4	0.3–4.2
Posterior cortical	1	0.5	0.1–2.7
Posterior polar^a^	3	1.4	0.3–4.2
Posterior capsular	1	0.5	0.1–2.7
Perinuclear (lamellar)	1	0.5	0.1–2.7

^a^ Occurred in the same eye of 1 horse.

^b^ Occurred in the same eye of 2 horses.

**TABLE 4:** Results of logistic regression examining the effect of age on cataract formation at the horse and eye levels with odds ratios (OR), 95% confidence intervals (CI) and *p* values.

	Horse level for 104 horses					Eye level for 208 eyes				
	Cataracts					Cataracts				
Age	Yes	No	OR	95% CI	*p*	Yes	No	OR	95% CI	*p*
<8 years	1	37	Ref.		0.02	2	74	Ref.		0.03
8–13 years	4	34	4.4	0.5–40.9	0.2	5	71	2.6	0.3–24.7	0.4
>13 years	8	20	14.8	1.7–126.9	0.01	12	44	10.1	1.2–85.9	0.03
Totals	13	91				19	189			

*Note*: Cataracts were not significantly more likely in the oldest horses compared with 8–13 year old horses (OR = 3.3, *p* = 0.07).

**FIGURE 4:**
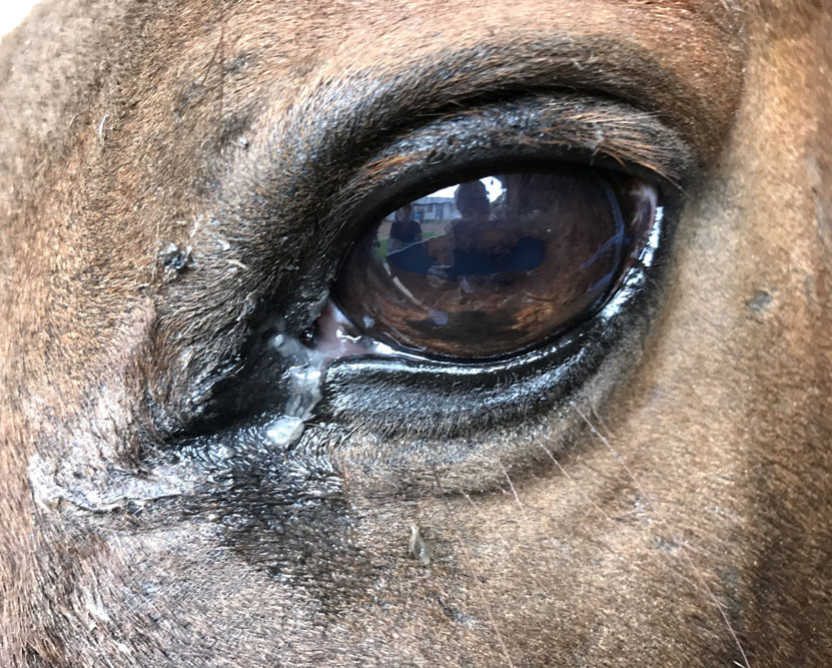
Mucopurulent discharge and epiphora with conjunctivitis.

**FIGURE 5:**
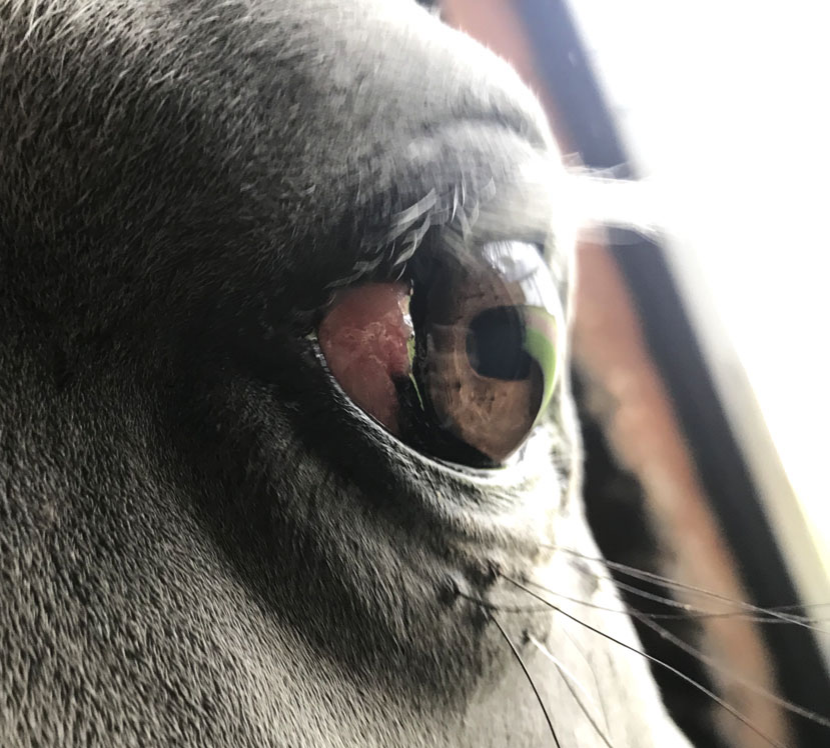
Proliferative, pink, lateral limbal mass.

**FIGURE 6:**
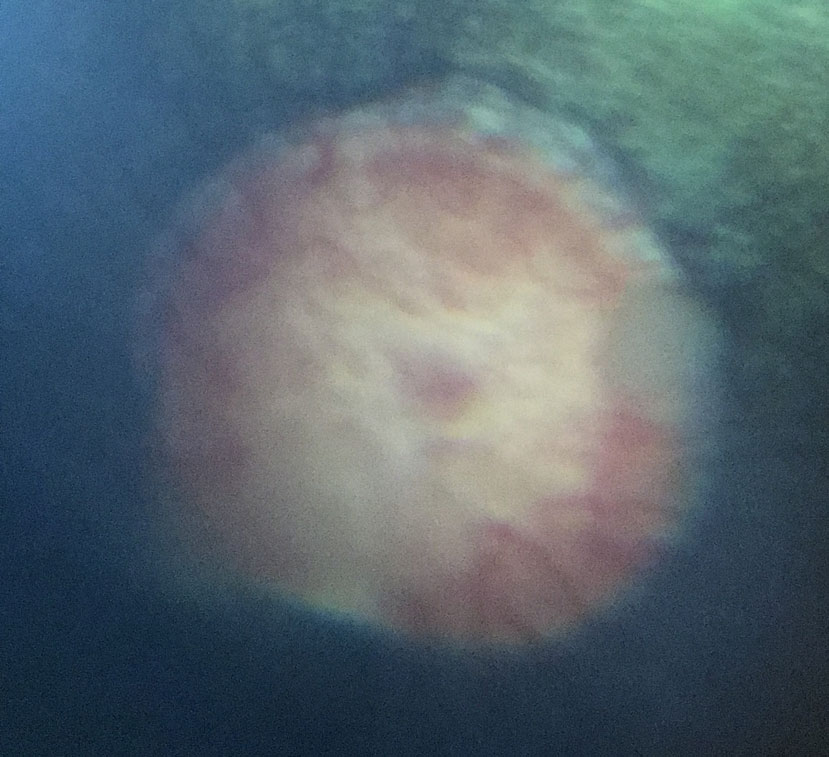
Focal proliferative change to the peripheral optic nerve head.

Eyelid pathology was uncommon with five eyes (2.4%) of five horses (4.8%) being affected. Pathology noted included eyelid fibrosis in two horses, an eyelid mass in two horses and a single chalazion in one horse. Similarly, few horses had conjunctival lesions with 15 eyes affected (7.2%) and eight horses affected (7.7%). Of the conjunctival pathology noted, conjunctivitis was the main lesion noted which was bilateral in five horses (Figure 4). Conjunctival follicles were noted bilaterally in one of these horses and one other horse had a unilateral conjunctival mass (Figure 5). Corneal lesions were uncommon too with three eyes (1.4%) and three horses affected (2.9%). Lesions included were corneal fibrosis in two eyes and a linear keratopathy in one eye. Corneal ulceration was not identified in any of the horses. Vitreal findings were noted in 13 eyes (6.3%) and 10 horses (9.6%). Vitreal degeneration was noted in eight eyes, asteroid hyalosis in four eyes and vitreal herniation in one eye. A single eye had optic nerve head changes resembling either an optic nerve mass (e.g., astrocytoma) or proliferative optic neuropathy (Figure 6).

Mean STT‐1 readings were 24.4 and 24.8 mm/min for the right and left eye, respectively, with a range of 8–35 mm/min (*p* = 0.7). Mean intraocular pressure (IOP) readings were 37 and 35 mmHg for the right and left eyes, respectively, with a range of 16–70 mmHg (*p* = 0.3). There were no statistical differences among right and left eye, sex or age categories for STT (*p* = 0.6 and 0.05) and IOP (*p* = 0.7 and 0.2) values.

## 4 DISCUSSION

This is the first report to the authors' knowledge investigating ocular findings in Warmblood horses in South Africa. Abnormalities reported were classified by area of the eye affected not on the presence of vision as in previous studies.^2^, ^14^ The highest prevalence of abnormalities in this study were chorioretinal following by iris and lens lesions. The presence of any chorioretinal lesions, and specifically focal chorioretinal lesions, were more likely with increasing age. Cataracts were also more likely to occur with increasing with age. However, due to the small sample size and low numbers of lesions, the confidence intervals for prevalence and the odds ratios for the effect of age are wide, indicating that there is substantial uncertainty in the effect estimates. Readers should interpret these results with caution.

Chorioretinal scarring was a frequent finding in this study which was seen as hyperreflective areas in the tapetal fundus and/or depigmentation or pigment clumping. Lesions were divided into peripapillary, non‐peripapillary, focal and linear chorioretinopathies to distinguish between both location and appearance of these lesions. All lesions noted were chronic and presumed to arise from previous inflammatory episodes, with less likely causes being trauma, equine recurrent uveitis (ERU) and manifestation from systemic disease.^15^ Focal chorioretinopathy, also known as ‘bullet‐hole’ chorioretinitis or chorioretinopathy, was the most commonly reported lesion overall with lesions appearing as small, white foci with a pigmented centre located ventral to the optic disc. The cause of bullet hole chorioretinopathy remains unclear with proposed aetiologies including ischaemia, inflammation and infarction^16^ and experimental infection with equine herpes virus (EHV‐1) in foals has been shown to induce similar lesions.^17^ The effect of bullet hole chorioretinopathy on vision has drawn much attention and speculation as some horses have larger numbers of foci which prompted a study by Allbaugh et al.^18^ who assessed outer retinal function in horses with extensive bullet‐hole chorioretinitis. This study showed that outer retinal function did not appear to be compromised. The current study showed 52.9% of horses had bullet‐hole lesions which was comparable with Hurn and Turner^2^ who reported a prevalence of 52.5% in racing Thoroughbreds; however, their population was significantly younger. These lesions have been reported with variable prevalence in other breeds such as 19% in Icelandic horses, 23% in Exmoor Ponies and 39% in Lipizzaners.^3^, ^7^, ^9^ Interestingly, two previous studies reported the number of bullet hole lesions as more or less than 20 bullet‐hole lesions which prompted the authors to record this data too. Hurn and Turner^2^ reported that 2.5% of their population were affected with greater than 20 bullet‐hole lesions whereas Rushton et al.^3^ reported that none were affected with large numbers of such lesions. In the current study only two horses were affected with such extensive ‘bullet‐hole’ lesions (1.9%). It must be noted that not all horses in previous studies were pharmacologically dilated and therefore bullet‐hole lesions should easily have been missed. Interestingly, the number of bullet holes increased with age in our study which was statistically significant, and it could be proposed that bullet hole lesions are a normal senile change. This is, however, in contrast to previous studies in geriatric horses and ponies which reported a prevalence of 3.6 and 8% in studies by Chandler et al.^19^ and Chalder et al.,^20^ respectively. Given these contradictory findings, more research is required to determine the aetiology of such lesions and determine if other factors such as genes and environment pay a role but overall it appears that these lesions do not appear to have a significant effect on vision.

Six horses (5.8%) in this study had peripapillary chorioretinopathy lesions also known as butterfly lesions which are seen as circumpapillary depigmentation and pigment clumping. This is similar to that reported in geriatric horses and ponies in a study by Chandler et al.^19^ Other similar studies generally reported lower numbers or the absence of ‘butterfly’ lesions altogether.^2^, ^3^, ^7^, ^8^, ^11^ Butterfly lesions are more commonly a unilateral finding; however, a single horse in our study had bilateral lesions. The cause of these inactive chorioretinal lesions are thought to arise from previous inflammation and have been reported with a prevalence of 5% in normal, healthy horses.^19^, ^20^ Previous studies^21^, ^22^, ^23^, ^24^ have speculated the involvement of ERU in peripapillary lesions; however, this relationship is not well understood. A newer study in Appaloosa horses demonstrated no significance between the presence of butterfly lesions and ERU^25^ which most likely indicates that other causes of inflammation are involved such as systemic disease or blunt trauma. The latter has been demonstrated in a study by Charnock et al.^26^ in which 29.1% of confirmed cases of blunt ocular trauma resulted in butterfly lesions. Butterfly lesions are, however, thought to have a more significant effect on vision due to their proximity to the ONH and a significant number of RGC axons.^27^

Non‐peripapillary lesions included non‐raised areas of tapetal hyperreflectivity or non‐tapetal depigmentation with pigment clumping which varied in size but were generally half to one optic nerve head diameter. Some of these lesions were consistent in appearance with early senile retinopathy or may represent a previous inflammatory episode. A further three eyes of three horses also had ‘linear chorioretinopathy’ lesions, the cause of which is unknown and not reported elsewhere but may represent previous focal retinal detachment or aberrant helminth migratory tracts.

Iridial lesions were the second most common in our study with the vast majority being diffuse iris hyperpigmentation. While it is widely accepted that iris hyperpigmentation can be associated with chronic inflammation, only one horse showed evidence of previous ocular trauma and inflammation as shown by a torn granula iridica, posterior synechiae and cataract formation. A study by Andrysikova et al.^8^ showed that iris hyperpigmentation statistically increased with age (*p* = 0.01) and in the current study horses which were affected bilaterally were older than those affected unilaterally; however, this was not statistically assessed.

Cataractous lesions were the third most common lesion in our study with an overall prevalence comparable to that reported for breeds which have been surveyed such as the Lipizzaner, Polish Arabian, Old Kladruber Horses and Miniature Horses.^3^, ^6^, ^8^, ^10^ The most common cataract noted in this study was an anterior capsular cataract followed by an axial (floriform) cataract, both of which are thought to be non‐progressive.^13^ However, the only way to monitor progression would be sequential examinations which was beyond the scope of this study. Five out of 13 horses affected by cataracts were affected bilaterally (38.5%), of which four of the five were symmetrical which may suggest a congenital or hereditary link such as in other breeds,^5^, ^28^, ^29^, ^30^ but further research would be required. Overall, the prevalence of cataract increased with age, which is supported by other studies with both Ireland et al.^31^ and Chalder et al.^20^ demonstrating a greater than 50% prevalence in their geriatric equid populations assessed.

Overall, very few eyelid, conjunctival and corneal lesions were reported. Most conjunctival lesions noted in this study were conjunctivitis which was mainly bilateral with conjunctival follicles also seen bilaterally in a single horse. This is in comparison to breeds such as the Lipizzaner^3^ and Icelandic horses^9^ which showed a large number of horses with conjunctival abnormalities. Several horses with conjunctivitis in our study were stabled at the same farm, which was situated on a dry, dusty hilled area and therefore an allergic aetiology is speculated but other factors such as housing, nutrition and infectious causes can play a role.

Corneal lesions also had a low prevalence in contrast to other studies. Rushton et al.^3^ noted a high percentage (21.7%) of corneal opacities in Lipizzaner horses and speculated an underlying immune‐mediated process in the Lipizzaner breed. Our findings are similar to that of Hurn and Turner^2^ who also demonstrated a low prevalence of corneal pathology in Thoroughbred horses.

Schirmer tear test data in this study are in line with data recorded by Beech et al.^32^ who also found no difference between left and right eyes, and with age having no significant effect. This is, however, in contrast to findings by Piccione et al.,^33^ who found a significant difference between left and right eyes, as well as between sexes.

The mean IOP readings in this study were substantially higher than the acceptable normal reference range of 12–30 mmHg.^34^ Our findings were also substantially higher than previous publications using rebound tonometry.^14^, ^21^, ^35^ Some values were as high as 50–70 mmHg but in the absence of clinical signs of glaucoma or vision loss, the authors feel this should be interpreted with caution as various factors can influence IOP such as head and body position,^36^, ^37^, ^38^ restraint,^39^ sedation,^40^, ^41^, ^42^, ^43^, ^44^ nerve blocks,^45^, ^46^ and diurnal variation can affect readings.^47^ The raised IOP values in this study may have resulted from all horses being examined conscious, without sedation and the use of nerve blocks, with different head positions and at various times of the day. This is a limitation of the current study as the authors are unable to draw any conclusions from this data set. Horses were not sedated during examination due to time and financial constraints, and the authors felt this was unethical for the scope of the study.

Other limitations of the current study include the small size sample, limited geographic area and use of a convenience sample. Given that this sample is from a concentrated area and therefore horses may therefore be more prone to conditions with an environmental influence or may also be genetically related. Future studies would aim to assess larger number of Warmblood horses from a wider population. Furthermore, the authors wanted to eliminate any bias by removing patients with a history of ophthalmic conditions as the aim of the study was to assess ‘presumed’ normal eyes. This led to one horse being excluded due to previous enucleation secondary to trauma. Examination was also declined by some owners due to concerns for pharmacologic dilation.

In summary, this is the first report to the authors' knowledge reporting ocular findings in Warmblood horses. Despite all horses in this study being visual as assessed by menace response, the importance of a comprehensive ophthalmic examination cannot be underestimated during both routine and prepurchase examinations. Additionally, all horses should be pharmacologically dilated with a short‐acting mydriatic agent and assessed in a darkened stable as lesions can easily go undetected if this is not performed. Despite not presenting for not having any known history of ocular disease, a large percentage of horses had ocular pathology thereby highlighting the importance of comprehensive ophthalmic examination.

## FUNDING INFORMATION

No funding was received for this research.

## CONFLICT OF INTEREST STATEMENT

The authors declare no conflicts of interest.

## AUTHOR CONTRIBUTIONS

**Ramona Allen:** Conceptualization; investigation; writing – original draft; methodology; writing – review and editing; data curation; project administration; formal analysis; visualization; validation; software. **Antony D. Goodhead:** Conceptualization; investigation; writing – original draft; methodology; writing – review and editing; project administration; data curation; supervision; resources.

## DATA INTEGRITY STATEMENT

Ramona Allen had full access to all the data in the study and takes responsibility for the integrity of the data and the accuracy of data analysis.

## ETHICAL ANIMAL RESEARCH

This study was approved by the Animal Ethics Committee of the University of Pretoria, South Africa (reference no. V068‐17).

## INFORMED CONSENT

Written consent for ophthalmic examination was obtained from all horse owners or stable yard managers.

### PEER REVIEW

The peer review history for this article is available at https://www.webofscience.com/api/gateway/wos/peer-review/10.1111/evj.14427.

## Data Availability

The data that support the findings of this study are openly available in FigShare at https://figshare.com/account/items/25425139/edit.
